# Mental health service providers: Barriers in collaboration

**DOI:** 10.1192/j.eurpsy.2021.1062

**Published:** 2021-08-13

**Authors:** O. Shchedrinskaya, M. Bebtschuk, E. Snarskaya

**Affiliations:** Science, Moscow State Budgetary Health Care Institution “Scientific and Practical Center for Mental Health of Children and Adolescents named after G.E.Sukhareva of Moscow Health Department, Moscow, Russia, Moscow, Russian Federation

**Keywords:** quality of services, barriers, collobiration, Child Psychiatry

## Abstract

**Introduction:**

Collaboration between psychiatrists and psychologists (counsellors) is one of the key factors impacting efficiency of services in child and youth mental health. Despite the clear benefits, a teamwork approach is still limited and has some difficulties.

**Objectives:**

The objective of the study was to explore potential barriers in the collaboration between professionals with different backgrounds.

**Methods:**

Anonymous online survey for staff from various mental health clinics across Russia was completed by 142 psychologists and 70 psychiatrists (Σ =212).

**Results:**

77.7% participants reported that collaboration is helpful in adult mental health services; 91.3% see partnership as an essential part of child and youth mental health. 61.6% specialists work together; 44.7% described it as a successful experience. At the same time 58.4% believe that pharmacological treatment should start first, and counselling may be postponed. 49.5% believe that doctors often diminish the importance of counselling. Fears and biases towards psychiatrists were reported by 28.9% of the sample. 25.4% participants reported lack of trust and limited understanding of counselling methods. Top barriers for collaboration that were reported: lack of opportunities on an organizational level (20% doctors and 45% psychologists), unclear professional boundaries and responsibilities (28.5% doctors and 15.4% psychologists), lack of motivation (20% doctors and 7% psychologists), lack of positive experience (11.2% psychologist and 0% doctors). The main reported benefit of collaboration by 39.6% was improved compliance and better treatment outcomes.

**Conclusions:**

In order to make collaboration among mental health professional more efficient, there is a need to address the barriers listed above.

